# Suppression of Paroxysmal Atrial Fibrillation by Pacing

**Published:** 2003-04-01

**Authors:** Anoop K Gupta

**Affiliations:** Krishna Heart Institute, Ahmedabad, India

Atrial Fibrillation (AF) affects approximately five million people world- wide. The incidence of AF increases with aging and more common in males [1]. Atrial fibrillation is the most common cardiogenic cause of stroke and exacerbates heart failure. Despite the prevalence of AF, it is still one of the most difficult arrhythmia to treat [2]. The management option of AF ranges from pharmacological therapy, catheter based ablation and surgery. However, non-traditional uses of pacemaker therapy have been reported in recent past.

Pacing therapies for AF are specific to the nature of the patient's AF [3,4]. Pharmacological management alone is the typical initial course of action. In AF cases where rapid ventricular rates cannot be controlled by pharmacological therapy, ventricular pacing therapy may be combined with radiofrequency catheter ablation of AV node [5].

An atrial based pacing mode compared with ventricular pacing, results in better clinical and hemodynamic conditions for patients with ventricular dysfunction [6]. Rosenqvist M et al [7] demonstrated that ventricular based pacing is associated with abnormal activation (e.g., pre-excitation) patterns that adversely affect cardiac hemodynamics. Specially, cardiac output and peak ventricular filling rates were found to be significantly lower in ventricular paced groups as compared to atrial based pacing groups. As well, lower peak oxygen uptake levels and oxygen saturation levels were found in ventricular paced groups. Similarly, Vardas et al [8] compared DDD and AAI pacing modes and concluded that atrial based pacing, at rest, produces a significantly greater cardiac output as compared to the DDD mode. However, no differences were found between the pacing modes during increased heart rates.

Atrial overdrive pacing therapy for patients with paroxysmal AF is done with the intent of altering or controlling AF [9]. Initiation of AF can be affected by suppressing the atrial premature beats that typically triggers or by modifying their propagation. The mean heart rate just before the onset of an AF episode is slightly higher than at rest and is very difficult to observe in most instances. This makes AF difficult to predict and monitor using pacing technology. Consequently, continuous overdrive atrial pacing is proposed to be a good course of treatment. The theoretical basis for overdrive pacing is the idea to continuously pace the atrium at higher rate than the patient sinus rate, so that it could alter the intrinsic atrial rate, propagation and suppress automaticity, caused by electrical remodeling, in the diseased fibers [10].

The case report by Levine et al [11] in this issue has discussed the utility of this therapy in a symptomatic patient with additional sinus node dysfunction, who was intolerant to the pharmacological therapy. The patient was marked symptomatic with paroxysmal AF and medical therapy, which got remarkably subsided with DDD pacing and withdrawal of medicine, however, the episodes of paroxysmal AF reduced after atrial fibrillation suppression algorithm.

The AF suppression algorithm is a newly approved algorithm designed to provide a high percentage of atrial overdrive pacing, which control the atrial rate, either from the sinus node or an ectopic foci. The objective is reduced temporal dispersion of the atrial refractory period combined with overdrive suppression of ectopic beats, a common trigger for atrial tachyarrhythmias. Automatic mode switch (AMS) remains functional with the AF Suppression algorithm on, thus if AF occurs, AMS is activated facilitating management as this provides information as to the frequency and duration of each AMS episode.

While the algorithm may not be 100% effective in all patients, any reduction in the number of AF episodes is beneficial. The use of a pacemaker containing the AF suppression algorithm should be considered in all patients undergoing device implant for a symptomatic bradycardia, particularly when there is a prior history of atrial fibrillation or they are at risk of AF which is more likely when the indication for pacing is sinus node dysfunction.
